# Role of mandibular displacement and airway size in improving breathing after rapid maxillary expansion

**DOI:** 10.1186/s40510-014-0040-2

**Published:** 2014-04-29

**Authors:** Rosamaria Fastuca, Piero Antonio Zecca, Alberto Caprioglio

**Affiliations:** 1 Department Surgical and Mophological Sciences, University of Insubria, Varese 21100, Italy

**Keywords:** Maxillary expansion, Respiration, Mandible, Polysomnography, Cone beam computed tomography

## Abstract

**Background:**

Oral breathing and maxillary deficiency are often associated with steep mandibular plane angle, and retrognathic mandible compared with the faces of healthy controls. Some studies suggested that after rapid maxillary expansion, improvement in nasal breathing and repositioning of mandible with transitory increasing of facial height and, in some cases, spontaneous forward repositioning might occur. The abovementioned mandibular effects could contribute to enlarge oropharynx volume with repositioning of tongue and soft palate with an improvement of upper airway volume after treatment. The aim of this study was to investigate by cone beam computed tomography the role of oropharyngeal volume and mandibular position changes after rapid maxillary expansion in patients showing improved breathing pattern confirmed by polysomnography exam.

**Methods:**

The final sample of this retrospective study comprised 14 Caucasian patients (mean age 7.6 years) who undergone rapid maxillary expansion with Haas-type expander banded on second deciduous upper molars. Cone beam computed tomography scans and polysomnography exams were collected before placing the appliance (T0) and after 12 months (T1). Mandibular landmarks localization and airway semiautomatic segmentation on cone beam computed tomography scans allowed airway volume computing and measurements*.*

**Results:**

No significant differences were found between oropharyngeal airway changes and mandibular displacement after rapid maxillary expansion in growing patients.

**Conclusions:**

The suggested improvement in upper airway and breathing after rapid maxillary expansion should be further related to different compartments of airway such as rhinopharynx and nasal cavity.

## Background

Several studies investigated the relationship between breathing pattern and craniofacial morphology [1-5], but these connections are not still completely clarified. The existence of correlations between airway obstructions and frequency of malocclusions was frequently refuted [6,7].

Nevertheless, sagittal and vertical growth pattern seemed to be related to different breathing pattern and airway sizes. Significantly decreased nasopharyngeal volumes were reported using computed tomography (CT) in patients presenting mandibular retrusion when compared to the ones presenting mandibular prognathism [8] and also if compared to control group without retrognathism [9]. On the contrary, significantly higher oropharyngeal volume was reported in Class III malocclusion patients when compared to Class I patients [10].

Among the effects of rapid maxillary expansion (RME) treatment, improvements in breathing function were reported [11-13]. These changes were associated to reduce nasal obstructions [14] and effects in tongue position [15]. Transitory increasing of facial height and in some Class II patients even a forward relocation of the mandible might occur after RME [16,17]. The abovementioned mandibular effects could contribute to enlarge oropharynx volume with repositioning of tongue and soft palate with an improvement of upper airway volume after treatment. Recently, the lowering of radiation dose using cone beam computed tomography (CBCT) allowed the growth of several PC softwares which are manually either or automatically able to compute airway volumes in order to better understand changes after treatment. Moreover, functional data such as the ones obtained by rhinomanometric examinations and polysomnography (PSG), often employed in obstructive sleep apnea syndrome (OSAS) subjects, could be greatly helpful [13] as diagnostic tool and in measuring treatment outcomes.

The aim of the present retrospective study was to investigate by CBCT changes in oropharyngeal volume and mandibular position after RME in patients undergone breathing improvement confirmed by PSG exam.

## Methods

Ethical approval for this study was obtained from the local Ethical Committee (no. 5184), and informed consent forms were signed by the parents of all patients. The sample consisted of patients treated at the dental clinic.

The initial retrospective study sample comprised 23 Caucasian patients. Selection criteria were age 6 to 9 years, CVS 1 skeletal maturation, Class I, unilateral functional posterior crossbite, early mixed dentition, upper and lower first molars erupted, no systemic disease, no previous orthodontic treatment, no asymmetries, and breathing function improvement confirmed by PSG examination after treatment. Nine patients were considered dropout for low quality of the CBCT scans or because no improvement of PSG examination was noted after RME treatment. The final study sample comprised 14 patients (mean age 7.1 ± 0.6 years) who fully matched inclusion and exclusion criteria.

**Figure 1:**
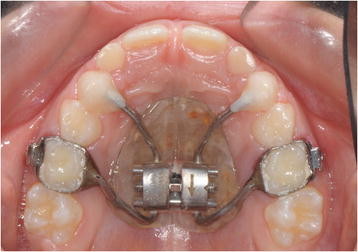
Haas-type expander on deciduous second molars.

The maxillary expander (Snap Lock Expander 10 mm. A167-1439, Forestadent, Pforzheim, Germany) used for all subjects was Haas-type expander banded to the upper second deciduous molars (Figure 1). The maxillary expanders were banded using a glass ionomer cement in accordance with the manufacturer's instructions. The screw of the palatal expander was initially turned two times (0.45 mm initial transversal activation). Afterwards, patients were instructed to turn the screw once per each following day (0.225 mm activation per day). The maxillary expansion was performed until dental overcorrection (2 mm) was achieved or when occlusion relationship evaluated at the first permanent molars was cusp to cusp. At the end of the active expansion period, the screw was locked with light-cure flow composite. The palatal expander was removed 12 months after it was inserted, at the end of the retention period. During this period, no other fixed orthodontic appliances were used in any patients.

CBCT scans (i-CAT, Imaging Sc. Int., Hatfield, PA, USA) were performed in seated position before inserting the maxillary expander (T0) and at the end of retention (T1), 12 months later when the expander was removed.

PSG examination (Embletta - EMBLA, Thornton, CO, USA) was performed for all subjects at T0 and T1 to collect oxygen saturation (SpO_2_) and apnea/hypopnea index (AHI).

**Figure 2:**
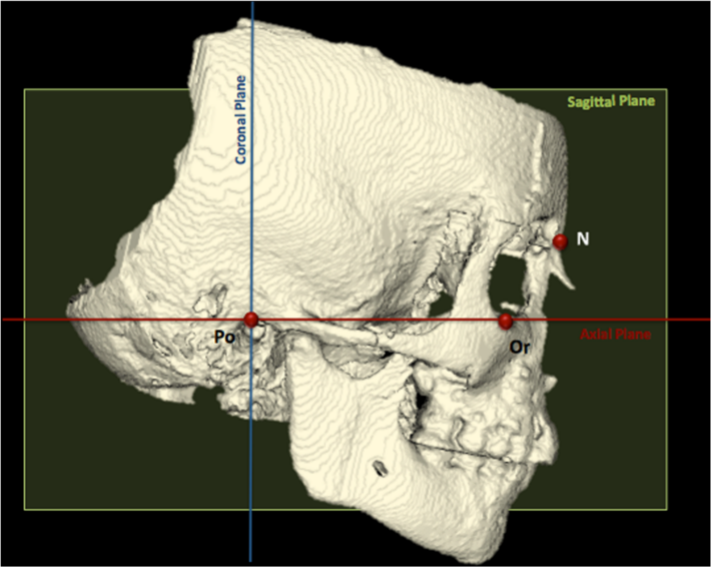
**Set of reproducible landmarks and planes to perform the reslice of head and reference system.** Po, porion right and left; Or, orbitale right and left; N, nasion. Axial plane (red), Frankfourt plane passing through PoR-PoL-OrR-OrL; coronal plane (blue), plane passing through PoR and PoL perpendicular to axial plane; sagittal plane (green), plane passing through N perpendicular to axial and coronal plane.

Dicom images were acquired in Mimics software (version 10.11, Materialise Medical Co., Leuven, Belgium). First, a set of reproducible landmarks and reference planes was defined to obtain a reproducible position of head (Figure 2).

**Figure 3:**
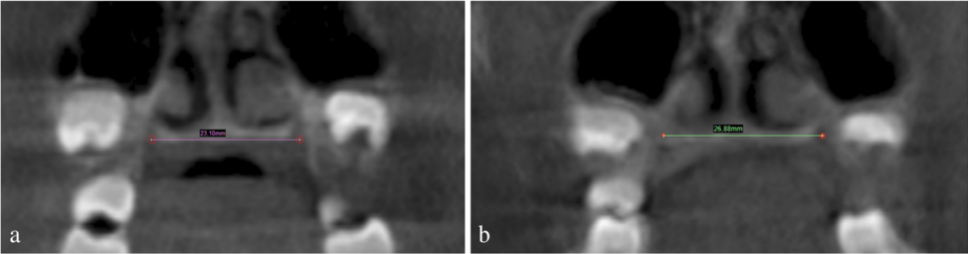
**Total expansion measured at the palatal foramens. (a)** Before RME. **(b)** After RME.

Palatal foramen right and left landmarks (PaFR-PaFL) were located, and the distance between them was used to assess the total amount of skeletal maxillary expansion (Figure 3).

**Figure 4:**
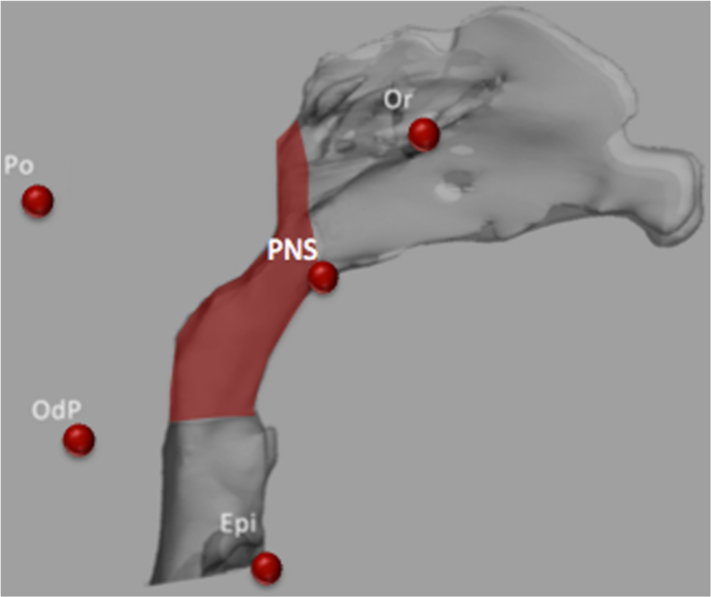
**Oropharyngeal airway volume segmentation.** PNS, posterior nasal spine; OdP, middle point of odontoid process of second cervical vertebra; Epi, epiglottis landmark.

The airway was segmented using thresholding-based segmentation manually corrected slice by slice. The upper limit of oropharyngeal airway was set as a plane passing through posterior nasal spine (PNS) and parallel to coronal reference plane defined as PNS plane. The lower limit was set as a plane passing through the middle point of odontoid process of second cervical vertebra (OdP) and parallel to axial reference plane defined as OdP plane. The limits of the oropharyngeal volume are shown in Figure 4.

Segmented airway and landmarks were then exported respectively in stereolitographic (.stl) and IGES (.igs) files. Segmentation and computing the airway is part of a previous study [13].

The oropharyngeal airway volume and landmarks files were imported in Rhinoceros Software (Robert McNeel & Associates, Seattle, WA, USA) where a logarithmic sequence built for this purpose automatically computed planes and oropharyngeal volume.

**Figure 5:**
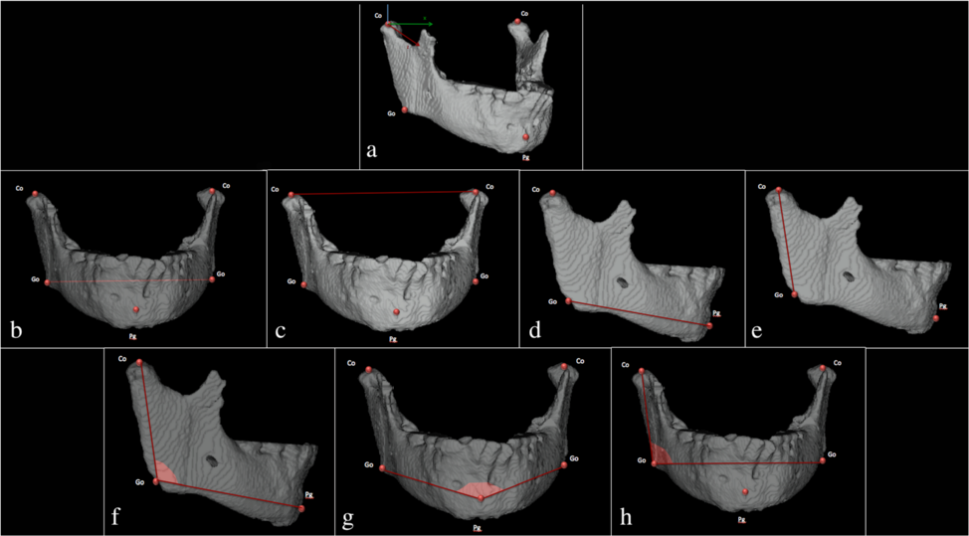
**Cephalometric analysis to evaluate mandibular displacements.** Co, condylion right and left; Go, gonion right and left; Pg, pogonion right and left. **(a)** Co *x, y, z* (mm), Co displacement on *x* (up-down displacements), *y* (right-left displacements), and *z* (anterior-posterior displacements) axis referred to the cranial reference system. **(b)** GoR-GoL, Go right and left width. **(c)** CoR-CoL, Co right and left width. **(d)** Go-Pg, Go-Pg width. **(e)** Co-Go, Co-Go width. **(f)** Co^Go^Pg*,* gonial angle. **(g)** GoR^Pg^GoL*,* Go right-Pg-Go left angle. **(h)** CoR^GoR^GoL, Co-Go, contralateral Go angle.

Mandible was then fully segmented from the DICOM images, and displacements of a set of mandibular landmarks were calculated using Mimics software performing dedicated 3D cephalometric system (Figure 5).

### Statistical analysis

The SPSS software, version 13.0 (SPSS® Inc., Chicago, IL, USA) was used to perform the statistical analyses. The Shapiro-Wilk test and Levene test confirmed the normal distributions and equal variances between T0 and T1, respectively. Means and standard deviations (SD) were computed for all the imaging and functional variables.

A paired sample *t* test was employed to assess the significance of the difference of each parameter between the time points. A *p* value < 0.05 was used in the rejection of the null hypothesis.

### Method error

The same trained operator (PZ) performed and repeated all measurements 1 month later. Systematic and random errors were calculated comparing the first and second measurements with paired *t* tests and Dahlberg's formula [18], at a significance level of *p* < 0.05. All measurement error coefficients were found to be adequate for appropriate reproducibility of the study.

## Results

Skeletal maxillary expansion, assessed as the PaFR-PaFL distance, showed an increase of 2.5 ± 0.2 mm (mean and SD), confirming the efficacy of RME treatment. Image analysis showed results regarding oropharyngeal volume and mandibular position changes.

Mean and SD for the two time points and results of paired *t* test are shown for oropharyngeal volume (Table 1) and mandibular position (Table 2). No significant differences were found in either cases. Oropharyngeal volume size underwent a slight increase (1,092.0 ± 776.9 mm^3^), but it did not reach the statistical level of significance (Table 1). Similarly, little changes were recorded in the displacement of mandible landmarks, but none of them was statistically significant (Table 2).

**Table 1:** Oropharyngeal volume at T0 and T1

**Variable**	**T0**	**T1**	**T1-T0**
Oropharyngeal Volume (mm^3^)	5,975.0 ± 2,423.7	7,067.5 ± 3,200.6	1,092.0 ± 776.9

Data are shown as mean ± SD. Levels of significance, *p* < 0.05.

**Table 2:** Mandibular displacements at T0 and T1

**Variable**	**T0**	**T1**	**Paired** ***t*** **test**
CoR_*x* (mm)	44.60 ± 2.32	44.88 ± 3.09	0.79
CoR_*y* (mm)	13.53 ± 3.75	13.33 ± 4.42	0.90
CoR_*z* (mm)	20.12 ± 3.69	20.72 ± 5.04	0.72
CoL_*x* (mm)	46.07 ± 2.48	46.82 ± 2.80	0.46
CoL_*y* (mm)	9.23 ± 3.06	5.86 ± 9.89	0.23
CoL_*z* (mm)	20.53 ± 2.34	20.12 ± 5.24	0.79
GoR_GoL (mm)	81.93 ± 4.67	82.32 ± 3.94	0.81
CoR_CoL (mm)	90.84 ± 4.01	92.00 ± 4.13	0.46
GoR_Pg (mm)	73.51 ± 4.98	75.75 ± 4.98	0.24
GoL_Pg (mm)	74.34 ± 4.47	76.02 ± 4.99	0.36
CoR_GoR (mm)	48.67 ± 4.24	49.81 ± 4.63	0.50
CoL_GoL (mm)	46.47 ± 5.15	49.21 ± 5.03	0.17
CoR_GoR_Pg (°)	120.03 ± 5.04	120.01 ± 3.78	0.99
CoL_GoL_Pog (°)	120.58 ± 4.05	120.08 ± 3.06	0.71
Go_Pg_Go (°)	67.44 ± 4.26	65.82 ± 4.17	0.32
CoR_GoR_GoL (°)	95.01 ± 3.92	95.62 ± 3.65	0.68
CoL_GoL_GoR (°)	95.93 ± 3.99	95.85 ± 3.40	0.95

L, left; R, right. Data are shown as mean ± SD. Levels of significance, *p* < 0.05.

On the contrary, all the patients in the present sample underwent breathing improvement according to PSG examination. SpO_2_ and AHI changes showed significant differences between the two time points (Table 3) with high level of significance (*p* < 0.01). SpO_2_ showed 6.2 ± 0.5% of increase and AHI significantly decreased of −4.3 ± 0.6 events.

**Table 3:** PSG examinations at T0 and T1

**Variable**	**T0**	**T1**	**T1-T0**
SpO_2_ (%)	89.8 ± 1.1	95.5 ± 1.6	6.2 ± 0.5**
AHI (events)	5.7 ± 1.2	1.4 ± 0.6	−4.3 ± 0.6**

Data are shown as mean ± SD. SpO_2_, oxygen saturation; AHI, apnea/hypopnea index. Levels of significance, *p < 0.05; **p < 0.01.

## Discussion

The objective of the present study was to use CBCT to investigate whether changes in oropharyngeal volume and mandibular displacement significantly influenced the breathing pattern improvement shown by PSG recordings in growing patients after RME treatment.

According to our results, all the patients of the present sample underwent an improvement of their breathing pattern according to the PSG examinations. SpO_2_ increased, meanwhile AHI decreased at T1 suggesting functional improvement in the breathing pattern because of the enhancement of the oxygen saturation and reduction of the apnea/hypopnea events. Similar results were reported by other studies [19] which investigated breathing performances in patients after RME using PSG examination in OSA patients and found significant improvements in AHI that remained stable after 24 months after treatment. Other authors evaluated the outcomes of RME treatment by means of functional examinations such as rhinomanometry (RMN) [12] and acoustic rhinometry (AR) [20,21] finding significant decrease in nasal airway resistance (NAR) with consequent improvement of nasal breathing.

Even though breathing improvement was recorded in the present study sample, no significant difference was found in oropharyngeal volume changes neither significant mandibular displacements. According to the present results, the functional breathing improvement did not seem related to hypothesize mandible repositioning and oropharyngeal volume enlargement. Previous studies investigated airway changes after RME analyzing different airway compartments or the whole volume [22-29].

El and Palomo [29] performed a morphological evaluation of airway volumes comparing RME-treated patients to a control group finding significant increases in nasal volumes. Nevertheless, neither oropharyngeal volume nor mandibular displacements underwent to significant changes after RME treatment according to their results. Zhao et al. [23] included an untreated control group and found no significant changes between treated and controls in retropalatal and retroglossal airways after RME treatment. The results of the present investigation are in agreement with the previously reported studies, and they suggest that the reasons of breathing improvement after RME in the investigated sample might not lie in oropharyngeal volume changes but rather should be researched in other compartments of airway such for example nasal cavity. According to other authors [29], the effect of RME on the upper airway might diminish farther down the airway and separating from the maxillary suture where the appliance forces are mainly exerted. A recent study [13] confirmed that nasal cavity volumes seem to be significantly influenced by RME unlike other compartments of airway.

Furthermore, the improvements of respiratory performances seem to be interestingly related more to the upper airway than to oropharyngeal airway in OSA patients who underwent maxilla-mandibular advancement within maxillofacial surgery [30].

Moreover, more complex mechanisms are involved in respiratory function changes after RME. Iwasaki et al. [15] recently compared changes of tongue posture with changes in the nasal airway ventilation pattern after RME treatment. According to their findings, children with nasal airway obstruction have a low tongue posture regardless of RME treatment meanwhile improvement of the nasal airway ventilation condition might be associated with improved low tongue posture after RME.

The measurement of the volumes of airway compartments may be biased by several factors such as head and tongue position during CBCT scan acquisition, breathing, swallowing movements, and repositioning of the tongue and the mandible after treatment [31]. Therefore, the reliability and repeatability of the CBCT recording of airway compartment has been questioned.

Several studies suggested mandible reposition after RME in Class II patients [16,17]. The sample of the present study did not include Class II patients but only Class I with positional posterior crossbite. The sample characteristics might have biased the present investigation considering that no forward repositioning of the mandible might occur in this study as it was shown in Class II patients. Nevertheless, mandibular shift might occur thus reducing the positional crossbite after RME. Previous studies [32] suggested small amount of changes in condylar position in patients presenting functional posterior crossbite and undergone RME. Moreover, the lack of a control untreated group, mainly for ethical reasons, also limits most of these studies. Furthermore, lacking of a control group does not allow to exclude that the slight recorded changes might be due to the growth during the observation time interval.

The present study was performed in a short-term period (1 year), and the short interval time might be biased by the results emphasizing the immediate changes around maxillary structures which are directly influenced by maxillary expansion. Long-term studies are further needed in order to investigate breathing pattern modifications and stability of the obtained results.

## Conclusions

Based on the result of this study, the following conclusions might be drawn:

 Oropharyngeal airway volume did not show significant changes before and after RME treatment.

 Significant mandibular displacements did not take place after RME in the investigated sample.

 PSG examinations revealed significant improvement of breathing function after RME which, then, seemed not related to an improvement of the oropharyngeal airway volume.

Neither oropharyngeal volume increase nor mandibular displacements seemed to have significant role in explaining the improvements in respiratory performance in the present study sample.

## Abbreviations

CT: Computed tomography: 

RME: Rapid maxillary expansion: 

CBCT: Cone beam computed tomography: 

SpO_2_: Oxygen saturation: 

AHI: Apnea/hypopnea index: 

RMN: Rhinomanometry: 

AR: Acoustic rhinometry: 

## Competing interests

The authors declare that they have no competing interests.

## Authors' contributions

RF drafted the manuscript. PZ acquired the clinical data and processed all the images for the analyses. AC supervised in acquiring clinical data, in drafting the manuscript and revising it critically for important intellectual content. All authors read and approved the final manuscript.
